# Multifunctional PVA–CMC/ZnO–Au Nanocomposite Films with Enhanced UV Shielding, Thermal Stability, and Antibacterial Performance

**DOI:** 10.3390/polym18060718

**Published:** 2026-03-16

**Authors:** Essam M. Abdel-Fattah, Ahmed M. Elnemr, Wafaa B. Elsharkawy, Tarek Fahmy

**Affiliations:** 1 Physics Department, College of Science and Humanities, Prince Sattam bin Abdulaziz University, Al-Kharj 11942, Saudi Arabia; 2 Polymer Research Group, Physics Department, Faculty of Science, Mansoura University, Mansoura ET-35516, Egypt; 3 Faculty of Health Science Technology, Mansoura National University, New Mansoura ET-35516, Egypt

**Keywords:** PVA/CMC, ZnO-Au nanoparticles, composite material, UV shielding, food packaging, optoelectronic material, antimicrobial activity, sustainable nanocomposites

## Abstract

Polyvinyl alcohol/carboxymethyl cellulose (PVA/CMC) blend nanocomposites reinforced with plasma-assisted synthesized zinc oxide–gold (ZnO–Au) nanoparticles were prepared via casting at varying nanoparticle concentrations. Structural and interfacial modifications were analyzed using XRD, FTIR, Raman spectroscopy, and XPS. XRD analysis confirmed the nanocomposite crystallinity, showing an average crystallite size of 24.48 nm and a lattice strain of 4.32 × 10^−3^ for the 0.15 wt% ZnO–Au composite. FTIR and Raman spectra revealed band shifts and broadening, indicating strong interactions between ZnO–AuNPs and the polymer matrix. XPS analysis further verified Zn and Au incorporation and changes in C 1s and O 1s intensities, reflecting modified surface chemistry. Optical analysis revealed a reduction in the band gap from 4.60 eV (pure PVA/CMC) to 3.52 eV for the 0.15 wt% ZnO–Au nanocomposite, accompanied by an increase in refractive index from 2.058 to 2.244, along with enhanced UV-shielding the performance due to reduced UV transmittance and increased film opacity. Thermogravimetric analysis demonstrated enhanced thermal stability, while antibacterial tests against E. coli and S. aureus confirmed strong antimicrobial activity. These findings demonstrate that PVA/CMC/ZnO–Au nanocomposites are a promising candidate for antibacterial, UV-blocking, food packaging, and optoelectronic applications.

## 1. Introduction

Polymer blending has emerged as an effective and versatile strategy for developing advanced materials with tunable properties. Unlike the time-consuming and costly synthesis of entirely new polymers, blending provides a faster and more economical route to achieving customized functionalities [1,2,3]. By adjusting the composition and molecular interactions, polymer blends can exhibit improved mechanical strength, thermal stability, and biological performance, making them increasingly valuable in a wide range of technological sectors [4,5].

Poly(vinyl alcohol) (PVA) is a synthetic, hydrophilic, and semi-crystalline polymer derived from the hydrolysis of poly(vinyl acetate) (PVAc) [6]. Its high-water solubility, biocompatibility, and resistance to many organic solvents make it an ideal material for use in packaging, adhesives, and biomedical applications [7,8,9]. When crosslinked, PVA can form hydrogels capable of absorbing large quantities of water, making it particularly useful in wound dressings and drug delivery systems [10,11,12].

Carboxymethyl cellulose (CMC), a water-soluble, biodegradable derivative of cellulose, has also gained attention as a sustainable natural polymer [13]. Its excellent film-forming capability, biocompatibility, and functional properties such as emulsification, adhesion, and water retention have led to extensive applications in food, pharmaceuticals, textiles, and electronics [14,15,16,17,18]. Because both PVA and CMC are hydrophilic and rich in hydroxyl groups, they form strong intermolecular hydrogen bonds, resulting in enhanced miscibility and film integrity. Consequently, blending PVA with CMC produces a hybrid film that combines the mechanical and structural stability of PVA with the flexibility and biodegradability of CMC, yielding a composite material with superior overall performance compared to the individual polymers [19].

Incorporating metal oxide nanoparticles (NPs) into polymers has enabled the development of multifunctional materials with improved optical, mechanical, and antimicrobial properties [20]. Zinc oxide nanoparticles (ZnO NPs) are widely recognized for their chemical stability, non-toxicity, strong UV absorption, and broad-spectrum antimicrobial activity [21,22]. Previous studies have shown that incorporating ZnO NPs into PVA–CMC matrices enhances their physicochemical properties [23] and improves the biological performance of related polymeric films [24]. Similarly, gold nanoparticles (Au NPs), due to their surface plasmon resonance (SPR) and excellent biocompatibility, have been extensively explored in optoelectronic and biomedical applications [25].

Combining ZnO with Au NPs provides a synergistic approach to enhance optical response and surface reactivity. ZnO contributes efficient UV absorption and photocatalytic activity, while Au NPs introduce plasmonic effects and promote electron transfer at the semiconductor–metal interface, thereby improving charge separation and reactive oxygen species (ROS) generation [26,27]. Although ZnO/polymer and Au/polymer nanocomposites have been widely reported [23,24,25], studies integrating hybrid ZnO–Au nanostructures as a single unified nanofiller within a biopolymer blend remain limited. To the best of our knowledge, the incorporation of plasma-assisted synthesized ZnO–Au hybrid nanoparticles into a PVA–CMC blended matrix has not been previously reported. The plasma-assisted route of ZnO-Au NPs offers a green, surfactant-free method for producing well-dispersed ZnO–Au nanostructures with clean surface chemistry and enhanced interfacial compatibility [28,29]. This strategy enables the fabrication of multifunctional films exhibiting simultaneously improved UV-blocking capability and antibacterial performance, representing a measurable advancement beyond monometallic ZnO or Au-filled polymer systems.

In this study, a novel nanocomposite film based on a PVA/CMC blend incorporated with plasma-assisted synthesized ZnO-Au nanoparticles is presented. The interaction mechanisms between the PVA-CMC matrix and ZnO-Au NPs were investigated using Fourier-transform infrared (FTIR) and Raman spectroscopy, while X-ray photoelectron spectroscopy (XPS) provided insights into elemental composition and chemical state. The optical and thermal behaviors were examined through UV-Vis spectroscopy and thermogravimetric analysis (TGA), respectively. The influence of ZnO-Au NPs incorporation on the antibacterial performance of the PVA-CMC matrix was systematically examined. This work demonstrates an efficient strategy for fabricating multifunctional biopolymer-based nanocomposites and provides deeper understanding of the synergistic effects of ZnO-Au NPs within PVA-CMC networks. The prepared nanocomposite films exhibit promising potential for applications in advanced biomedical, antimicrobial, and optoelectronic technologies.

## 2. Materials and Methods

All chemicals used in this study were of analytical grade. The ZnO-Au nanoparticles were synthesized in two sequential steps as detailed in [29]. First, ZnO nanoparticles were prepared via a hydrothermal method by dissolving 0.05 M ZnCl_2_ in 100 mL of deionized water, followed by adjusting the pH to 10 under continuous stirring. Cetyltrimethylammonium bromide (CTAB, 0.0028 M) was added as a surfactant, and the solution was transferred to a hydrothermal reactor and heated at 120 °C for 4 h. The resulting precipitate was collected by filtration, washed several times with DI water, and dried at 60 °C for 24 h. In the second step, Au NPs were deposited on the ZnO surface using a non-thermal atmospheric-pressure argon plasma. A 0.25 mM HAuCl_4_ solution was used as the Au precursor, and 0.4 g of the as-prepared ZnO nanoparticles were dispersed in 100 mL of this solution and allowed to equilibrate for 30 min. The suspension was then exposed to an argon plasma plume for 5 min to induce the reduction of Au^3+^ ions and promote the formation of Au nanoparticles on the ZnO surface. The formation of ZnO–Au nanostructures was indicated by a color change from white to rose, and the final product was obtained by filtration and drying, yielding a purple powder.

PVA with a molecular weight of ~12,000 g/mol (CAS number: 5-89-9002) is obtained from BDH Chemicals Ltd., Poole (UK), and CMC with a molecular weight of ~93,000 g/mol. (CAS number: 4-32-9004) is obtained from Polysci, Inc., Niles, IL (USA). Separate aqueous solutions of PVA and CMC were prepared to form the PVA/CMC polymer blend. First, 1 g of CMC powder was dissolved in 100 mL of DI water (1% *w*/*v*) under gentle magnetic stirring until a clear solution was obtained. Similarly, 1 g of PVA was dissolved in 100 mL of DI water (1% *w*/*v*) at an elevated temperature (~80 °C) with the addition of 1% (*v*/*v*) acetic acid to facilitate dissolution and obtain a homogeneous solution. Both polymer solutions were stirred separately and subsequently mixed at a 1:1 volume ratio, followed by continuous stirring for 3 h to ensure complete blending and compatibility. This process yielded a clear and uniform PVA/CMC polymer blend solution, which served as the primary polymer matrix.

**Scheme 1:**
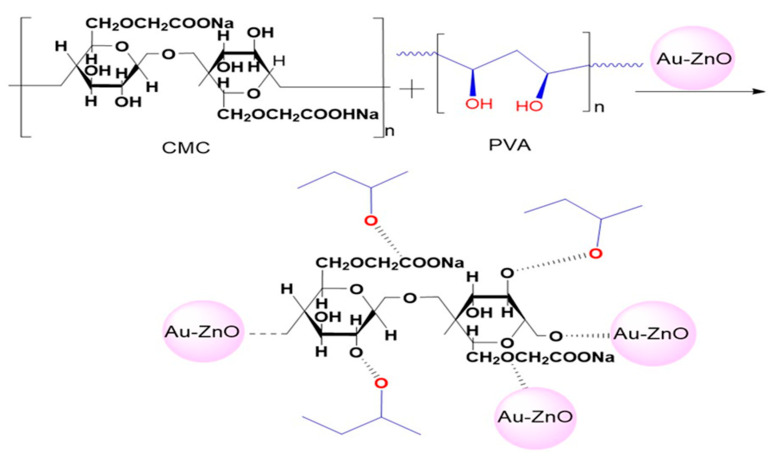
The suggested interaction between PVA, CMC, and ZnO-AuNPs.

Different loadings of ZnO–Au nanoparticles (0.01, 0.05, 0.10, and 0.15 wt%), calculated relative to the total polymer weight, were individually dispersed into predetermined volumes of the PVA/CMC polymer blend solution under continuous stirring to ensure uniform nanoparticle distribution. Each mixture was subjected to continuous magnetic stirring followed by ultrasonication for 30 min to ensure uniform dispersion of the ZnO-Au NPs within the PVA/CMC matrix. The resulting homogenous mixtures were then cast onto clean glass substrates and dried at 50 °C for 48 h to form thin films. The thickness of the prepared films was ~300 μm ± 5 μm, as measured using a digital micrometer. The proposed interaction mechanism between the PVA/CMC matrix and the ZnO-Au nanoparticles is illustrated in Scheme 1.

### 2.1. Film Characterization

XRD measurements are performed using a Bruker D8 advance powder XRD (Karlsruhe, Germany) with a CuKα radiation source (λ = 1.5418 Å at a current of 50 mA and voltage of 40 kV) and scan rate of 3°/min in the full range of measurements from 2θ = 5° to 70°. ATR-FTIR spectra are carried out for the samples in ambient air at room temperature in the range from 4000 to 300 cm^−1^ with a spectral resolution of 1 cm^−1^ using a Thermo Scientific iD5 ATR spectrometer (Yokohama, Kanagawa, Japan). Raman spectroscopy is performed in the range of 3500–300 cm^−1^ using Senterra II, Bruker (Edling, Germany). An atomic force microscope (AFM, Bruker, Billerica, MA, USA) equipped with an ultra-sharp probe was used to examine the surface morphology. The surface chemistry of the samples is investigated by the x-ray photoelectron spectroscopy XPS (Thermo K Alpha spectrometer, Waltham, MA, USA) using Al K alpha X-rays (1486 eV) with a spot size of 400 microns). UV/Vis measurements are carried out in the range from 200 to 900 nm using a UV-Vis spectrophotometer (UV-1601 PC, Shimadzu, Kyoto, Japan).

### 2.2. Antimicrobial Tests

The antimicrobial efficiency of pure PVA/CMC blend films and PVA/CMC/ZnO–Au nanocomposite films was evaluated using the standard agar disc diffusion method [30]. The foodborne pathogens *Escherichia coli* (Gram-negative) and *Staphylococcus aureus* (Gram-positive) were used as model organisms. The bacterial cultures were incubated at 37 °C for 16 h and subsequently diluted with sterile saline solution. The diluted suspensions were uniformly spread on nutrient agar plates. Circular film samples (4 mm diameter) were carefully placed on the inoculated agar surface, and the plates were incubated at 37 °C for 24 h. The antibacterial activity was assessed by measuring the diameter of the inhibition zones (mm) surrounding the film samples. All media and materials used during the procedure were sterilized by autoclaving to maintain aseptic conditions.

## 3. Results

### 3.1. Structural and Morphological Analysis

**Figure 1:**
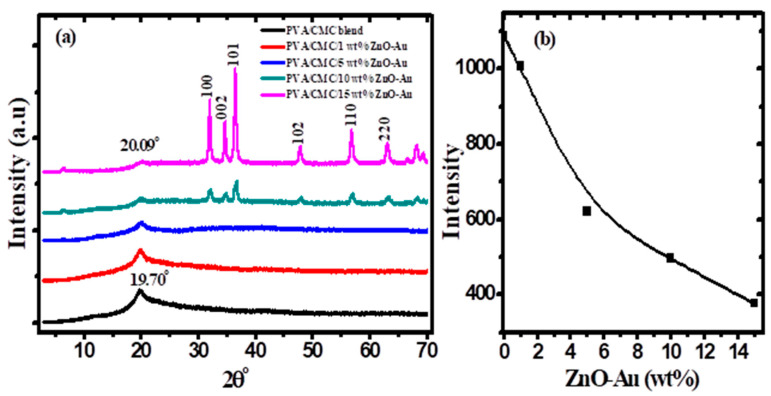
(**a**) XRD patterns of PVA/CMC/ZnO-Au nanocomposites, (**b**) variation of diffracted intensity at 2θ = 19.70° as a function of ZnO-Au wt.% loading.

The XRD pattern of the pristine PVA/CMC blend (Figure 1a) exhibits a characteristic broad diffraction peak at 2θ = 19.75°, confirming its semicrystalline nature. Upon incorporation of ZnO-Au NPs (Figure 1b), the intensity of the characteristic polymer peak at 2θ = 19.75° decreases significantly and shifts slightly to 2θ = 20.09°. This reduction in peak intensity, together with the slight shift, indicates disruption of intermolecular hydrogen bonding and partial loss of ordered chain packing within the polymer matrix. These structural changes are attributed to strong interfacial interactions between ZnO–Au nanoparticles and the hydroxyl (–OH) and carboxyl (–COOH) functional groups of PVA and CMC. Similar behavior has been reported in PVA-CMC-ZnO nanocomposites [31]. The PVA/CMC/0.15 wt% ZnO-Au nanocomposite pattern reveals additional sharp diffraction peaks at 32.13°, 34.79°, 36.64°, 47.86°, and 56.98°, corresponding to the (100), (002), (101), (102), and (110) planes of the hexagonal wurtzite structure of ZnO (JCPDS Card No. 96-900-4180) [32]. A distinct peak at 2θ = 63.34° is also observed, corresponding to the (220) plane of face-centered cubic (fcc) Au (JCPDS Card No. 96-901-1613) [33,34], further confirming the presence of Au nanoparticles with an estimated crystallite size of ~13 nm.

The calculated structural parameters such as average crystal size (*D*), lattice strain (ε), crystal number per unit area (*N*_c_), dislocation density (δ), and stacking fault (*SF*) are summarized in Table 1. These parameters were determined using the following relations [35]:

(1) $$D=\frac{0.94\lambda }{\beta \cos\theta }$$

(2) $$\varepsilon =\frac{\beta }{4\tan\theta }$$

(3) $$N_{c}=\frac{t}{D^{3}}$$

(4) $$\delta =\frac{1}{D^{2}}$$

(5) $$SF=\beta \left[\frac{2\pi }{45(\tan\theta {)}^{0.5}}\right]$$

The calculated structural parameters of the PVA/CMC/0.15 wt% ZnO–Au nanocomposite indicate a well-defined inorganic crystallite, with an average crystallite size of approximately 24.5 nm and a lattice strain of 4.32 × 10^−3^, indicating moderate internal stress within the nanocomposite matrix. The relatively low dislocation density (1.75 × 10^−3^ nm^−2^) and stacking fault value (1.16 × 10^−2^) confirm the high crystalline quality of the ZnO–Au NPs dispersed within the polymer matrix. The crystal number density (3.77 nm^−2^) further supports uniform NPs distribution. Therefore, while the incorporation of ZnO-Au NPs reduces the crystallinity of the polymer phase due to structural disruption, it introduces highly crystalline inorganic domains within the composite. This dual structural modification enhances interfacial interactions between the nanoparticles and the polymer network without implying an increase in polymer crystallinity.

**Figure 2:**
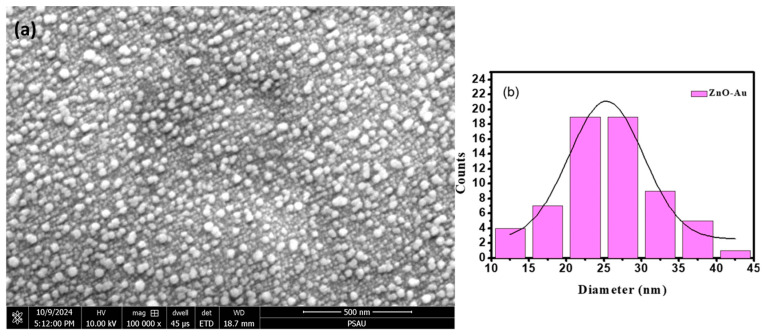
(**a**) SEM image of PVA/CMC/0.15 wt% ZnO-Au nanocomposite film and (**b**) particle size distribution of the incorporated nanoparticles.

The morphology of the PVA/CMC blend containing 0.15 wt% ZnO–Au NPs was examined using the SEM technique. As shown in Figure 2a, the SEM image reveals a uniform and homogeneous dispersion of ZnO–Au NPs throughout the PVA/CMC matrix, confirming their successful incorporation into the polymer network without noticeable agglomeration. The sample with the highest ZnO–Au loading ratio (0.15 wt%) was intentionally selected for SEM analysis because it represents the most critical condition for evaluating nanoparticle dispersion within the polymer matrix. At higher filler concentrations, nanoparticle aggregation is more likely to occur due to stronger particle–particle interactions. Therefore, confirming homogeneous dispersion at this highest loading level provides strong evidence of the effectiveness of the preparation method in preventing nanoparticle agglomeration.

The observed nanoparticle size in the SEM micrograph is consistent with the crystallite size estimated from XRD analysis. As illustrated in Figure 2b, the average diameter of the dispersed ZnO–Au nanoparticles is ~25 nm, indicating good compatibility and interfacial interaction between the nanoparticles and the polymer blend.

### 3.2. Spectroscopic and Composition Analysis

**Figure 3:**
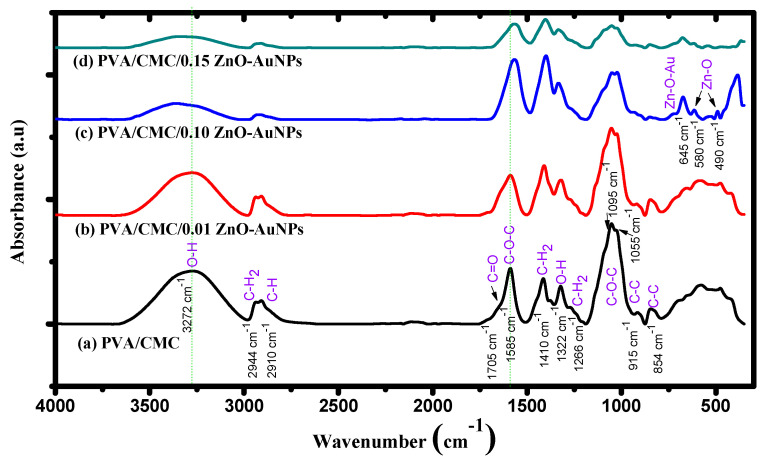
FTIR spectra of the pristine PVA/CMC blend and PVA/CMC composites incorporated with ZnO–AuNPs at different loadings.

Figure 3 shows the FTIR spectra of the pure PVA/CMC blend and the PVA/CMC films incorporated with varying weight percentages of ZnO-Au nanoparticles. The FTIR spectrum of the pure PVA/CMC film exhibits the characteristic absorption bands of both PVA and CMC. A broad band around ~3272 cm^−1^ is attributed to O–H stretching vibrations arising from hydroxyl OH groups in both polymers [36,37]. The band at 2944 cm^−1^ corresponds to C-H and CH_2_ stretching vibrations in the PVA and CMC backbone [38]. The band at 1585 cm^−1^ corresponds to the asymmetric stretching of the carboxylate (COO^−^) groups in CMC [39], while a weak shoulder observed at 1705 cm^−1^ is attributed to the C=O stretching of ester groups in PVA. The simultaneous presence of C=O (from PVA) and COO^−^ (from CMC) bands confirms the good miscibility molecular interaction between the two polymers. Additional characteristic bands appear at 1410, 1322, and 1266 cm^−1^, corresponding to CH_2_ scissoring, O–H bending, and CH_2_ bending modes, respectively [40]. Peaks at 1095 and 1055 cm^−1^ are attributed to C–O stretching in CMC and C–O–C (ether) stretching in the polysaccharide backbone [41]. The bands at 915 and 854 cm^−1^ are assigned to C–C skeletal vibrations, particularly from the PVA backbone [42].

The incorporation of ZnO-Au NPs into the PVA/CMC matrix markedly affects the polymer functional groups, indicating strong interfacial interactions between the nanoparticles and the polymer chain [14]. These interactions are reflected by distinct shifts in band positions and variations in band intensities with increasing ZnO-AuNP content. A noticeable blue shift in the O–H stretching vibration is observed, moving from 3272 cm^−1^ in the pure PVA/CMC blend to 3361 cm^−1^ after ZnO-Au NP incorporation. Concurrently, the band intensity decreases and broadens, suggesting a modification of the hydrogen bonding network within the polymer matrix. Similarly, the C=O stretching vibration of the carboxylate group in CMC exhibits a red shift from 1585 cm^−1^ to 1565 cm^−1^, with reduced intensity, implying coordination between the nanoparticle surface and carboxyl functionalities. The O–H bending vibration also experiences a blue shift from 1322 cm^−1^ to 1337 cm^−1^ and a gradual loss of intensity, further confirming interactions between the polymer hydroxyl groups and ZnO-Au NPs. These spectral modifications in the O–H, COO^−^, and O–H bending regions collectively indicate enhanced hydrogen bonding between the CMC and PVA chains, facilitated by the embedded ZnO-Au NPs. Additionally, the gradual disappearance of the C=O absorption band can be attributed to the decomposition of vinyl acetate groups in PVA under mildly alkaline conditions, leading to their substitution by hydroxyl groups and, consequently, the loss of the C=O band.

A noticeable decrease in the intensity of the CH_2_ asymmetric stretching band is observed with increasing ZnO-Au NP content. Since hydroxyl groups act as electron donors, the resulting increase in electron density around the carbon atoms bonded to OH groups likely affects neighboring methylene groups, thereby reducing the corresponding absorption intensity. In the low-frequency region (400–900 cm^−1^), several bands corresponding to Zn–O vibrations are observed [43,44], confirming the successful incorporation of the ZnO-Au NPs into the polymer network.

**Figure 4:**
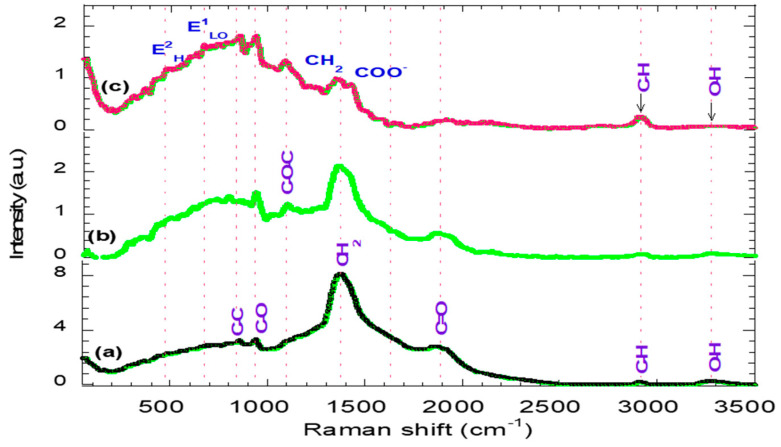
Raman spectra of (**a**) pristine PVA/CMC film and PVA/CMC nanocomposite films containing ZnO-AuNPs at different loadings: (**b**) 0.05 wt% and (**c**) 0.15 wt%.

Raman spectroscopy is a non-invasive technique used to investigate the structural composition of blended PVA/CMC films and to study the effect of varying ZnO-Au NPs weight percentages on their vibrational characteristics. The Raman spectrum of the pristine PVA/CMC blend film is shown in Figure 4a. A dominant strong peak at 1440 cm^−1^ is observed, which is attributed to the CH_2_ bending/scissoring modes of the PVA and CMC blend. Other smaller peaks are evident, including those at 853–937 cm^−1^ (C–C stretching), 1860 cm^−1^ (C=O stretching of residual acetate carbonyl from hydrolysis), 2910 cm^−1^ (C–H stretching), and 3280 cm^−1^ (O–H stretching) [45]. The shift in the C=O peak position is likely due to cross-linking interactions between the blended polymers.

The Raman spectra of PVA/CMC films incorporated with varying amounts of ZnO-AuNPs exhibit the same characteristic Raman modes as the pristine PVA/CMC blend but show changes in their relative intensities and band broadening. The incorporation of ZnO-Au NPs is confirmed by the appearance of small peaks at 490 cm^−1^ and 650 cm^−1^, corresponding to the E_2_(high) and E_1_(LO) phonon modes of ZnO [29]. Additionally, a new band emerges at 1099 cm^−1^, and the CH_2_ band exhibits splitting, which is likely due to the presence of AuNPs and their plasmonic effects, confirming the successful incorporation of the NPs and enhanced cross-linking interactions [45]. The band at 1099 cm^−1^ is assigned to C–O–C stretching vibrations, while the new shoulder at 1425 cm^−1^ near the CH_2_ band is attributed to the COO^−^ group. The reduction in intensity and broadening of the CH_2_ band in PVA/CMC/ZnO-Au nanocomposite films may be attributed to the incorporation of Zn and Au ions, which potentially replace some carbon atoms within the polymer matrix. As reported in [29], the presence of AuNPs on the surface of ZnO scatters the laser beam, leading to an overall reduction of Raman band intensities as observed in Figure 4b,c.

**Figure 5:**
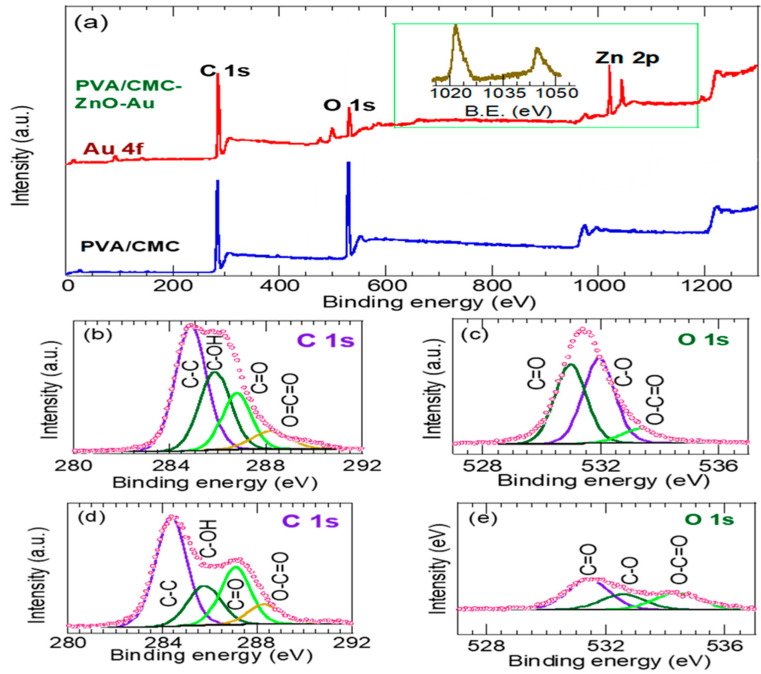
(**a**) XPS survey spectra and high-resolution C 1s (**b**,**d**) and O 1s (**c**,**e**) of PVA/CMC and PVA/CMC nanocomposites containing 0.15 wt% of ZnO/Au NPs.

The XPS survey spectra of the pure PVA/CMC blended film and the PVA/CMC composite film incorporated with 0.15 wt% of ZnO-AuNPs are presented in Figure 5a. The spectrum of the pure PVA/CMC blend revealed two prominent peaks at approximately 285 eV and 532 eV, corresponding to the C 1s (carbon) and O 1s (oxygen), respectively. In contrast, the XPS survey spectrum of the PVA/CMC/0.15 wt% ZnO-Au nanocomposite displayed additional features, including a small peak at ~84–87 eV attributed to Au 4f (gold) and a doublet within the binding energy range of 1020–1050 eV (inset of Figure 5a), associated with Zn 2p (zinc), that demonstrates the incorporation of ZnO/Au NPs in the PVA/CMC composite film [29].

To identify the various bonds present in the examined films, high resolution spectra of C 1s and O 1s are shown in Figure 5b–e. The C 1s spectrum of PVA/CMC blended film (Figure 5b) was deconvolved into four sub-peaks: ~284.88 eV (C–C/C–H, representing the polymer backbone), ~286.3 eV (C-OH/C-O, corresponding to hydroxyl and ether groups), ~287.1 eV (C=O, indicating carbonyl from CMC), and 288.3 eV (O-C=O associated with carboxymethyl of CMC) [46]. Upon the incorporation of ZnO-Au NPs, the C-OH/C-O component of the C 1s spectrum decreased, as depicted in Figure 5d, suggesting enhanced cross-linking within the polymer matrix. The ZnO-AuNPs likely interact with the C-OH groups in PVA and CMC, leading to this reduction. This observation is consistent with Raman results (Figure 4b,c), which show a reduction in O-H group intensity upon ZnO-AuNP incorporation into the PVA/CMC blended film.

The deconvolution of the high resolution O 1s spectrum for the PVA/CMC film is shown in Figure 5c. Three distinct sub-peaks are observed at: ~530.9 (C=O), ~531.98 eV (C-OH/C-O), and ~533.4 eV (O-C=O), corresponding to carbonyl, hydroxyl/ether, and carboxyl groups, respectively [47]. Incorporation of ZnO-AuNPs into the PVA/CMC blended film significantly reduces the intensity of oxygen-based groups, particularly the C-O/C-OH component, as seen in Figure 5d. This suggests that the oxygen-based groups form bonds with the ZnO-AuNPs, which impacts the physiochemical characteristics of the composite film. Notably, the O-C=O component shows a slight increase or remain unchanged upon ZnO-AuNPs incorporation, as shown in Figure 5e.

### 3.3. Optical Properties

**Figure 6:**
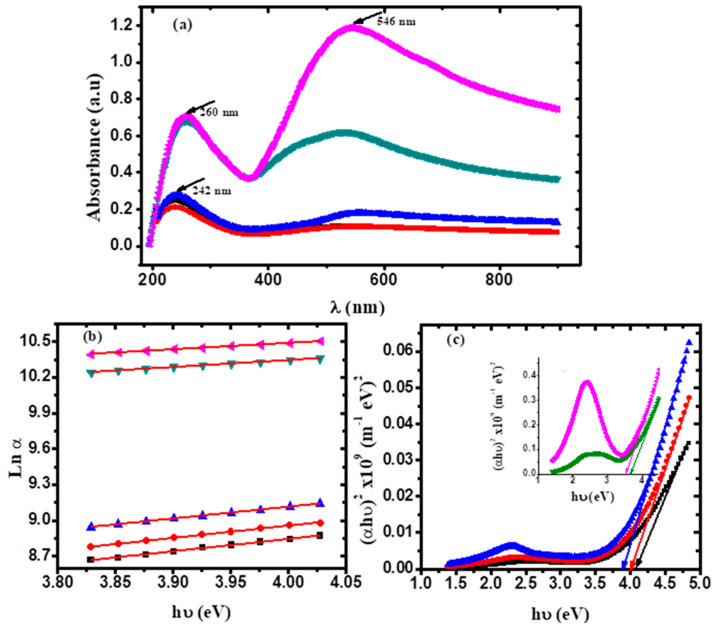
(**a**) The absorbance versus wavelength, (**b**) Ln α versus hυ, and (**c**) (αhυ)^2^ versus hυ for (■) PVA/CMC, (●) PVA/CMC/0.01 wt%ZnO-AuNPs, (▲) PVA/CMC/0.05 wt%ZnO-AuNPs, (▼) PVA/CMC/0.10 wt%ZnO-AuNPs, and (◄) PVA/CMC/0.15 wt%ZnO-AuNPs.

Figure 6a presents the UV-vis absorption spectra of the pristine PVA/CMC blend and PVA/CMC/ZnO-Au nanocomposite films in the wavelength range of 200–900 nm. The pure PVA/CMC blend exhibits a characteristic absorption peak at 242 nm, which is attributed to π–π* electronic transition of the polymer backbone. After incorporating ZnO-Au NPs, the absorption intensity increases notably, and the main peak shifts to ~260 nm, indicating enhanced absorption in the UV region. In addition, a distinct band appears around 546 nm, corresponding to the surface plasmon resonance (SPR) of Au NPs [29]. The appearance of this SPR band confirms the presence of Au NPs and contributes to improved visible-light interaction.

To further understand the structure disorder induced by ZnO-Au NPs incorporation, the Urbach energy (*E*_u_) was evaluated using the Urbach relation $\alpha =\alpha_{o}e^{\left(h\nu /E_{u}\right)}$, where α is the absorption coefficient (α = 2.303 *A*/*d*), $\alpha_{o}$ pre-exponential factor, and hν is the photon energy [48]. The plots of *Ln*α versus *h*υ for the investigated films are presented in Figure 6b. The $E_{u}$ values were obtained from the slopes of the liner regions and are listed in Table 2. The results reveal that *E*_u_ increases from 0.96 eV to 1.87 eV with increasing ZnO-Au NPs content, indicating increased structure disorder and defect density within the polymer matrix. This increased structural disorder contributes to the broadening of absorption edges and improved light attenuation. for allowed direct transition [49]. The corresponding (hν.α)^2^.

The optical band energy gap ($E_{g}$) was determined using Tauc’s relation ${\left(h\nu .\alpha \right)}^{2}=B\left(h\nu -E_{g}\right)$ for allowed direct transition [49]. The corresponding plots of (hν.α)^2^ versus photon energy (hυ) are presented in Figure 6c. The $E_{g}$ values were obtained by extrapolating the linear regions of these plots to the energy axis and are summarized in Table 2. The band gap decreases from 4.60 eV for pristine PVA/CMC to 3.52 eV for the highest ZnO–Au loading. This reduction suggests the formation of localized energy states associated with ZnO–Au NPs, which facilitate electronic transitions within the nanocomposite films. The combined effects of band gap narrowing, defect state formation, and plasmonic interaction extend light absorption toward the near-UV-visible region as seen in Figure 6a. Similar behavior has been reported for PVA/ZnO [10] and PVA/PVP/CMC/ZnO nanocomposites [19].

The UV-blocking performance of the films was further evaluated from the UV transmittance calculated using $T(\lambda )=10^{-A(\lambda )}\times 100\%$ where A(λ) is the absorbance at wavelength. The average transmission values in the UVC (220–280 nm), UVB (280–320 nm), and UVA (320–400 nm) regions are summarized in Table 2, together with the film opacity, defined as $\frac{A_{600}}{l}$, where $A_{600}$ is the absorption at 600 nm and *l* is the film thickness [50]. The pristine PVA/CMC blend exhibits relatively high UV transmission, with $T_{UVC}$ = 64.2%, $T_{UVB}$ = 75.5%, and $T_{UVA}$= 85.2%, indicating limited intrinsic UV-blocking capability of the polymer matrix. This behavior is consistent with the large optical band gap ($E_{g}$ = 4.60 eV) and low structural disorder ($E_{u}$ = 0.96 eV), which allow most ultraviolet radiation to pass through the film [51]. With the incorporation of ZnO–Au NPs, a progressive reduction in UV transmittance is observed across all UV regions. At low ZnO-Au NPs loading (0.01 and 0.05 wt%), the UV transmission decreases slightly, reflecting partial attenuation due to increased light absorption and scattering by the dispersed nanoparticles. For example, $T_{UVC}$ decreases from 64.2% for the pure film to 55.7% for the 0.05 wt% nanocomposite, accompanied by a reduction in the band gap from 4.60 eV to 4.30 eV and a slight increase in Urbach energy from 0.96 to 1.01 eV. These changes indicate the introduction of localized defect states that broadens the absorption edge. A more pronounced improvement in UV shielding occurs at higher nanoparticle concentrations (0.10 and 0.15 wt%). In these films, the UV transmittance decreases significantly to ~24–29% in the UVC and UVB regions and ~38–39% in the UVA region, demonstrating strong attenuation of UV radiation. This enhanced UV-blocking capability can be attributed to several synergistic factors: (i) band gap reduction that shifts the absorption edge toward the near-UV region, (ii) increased structure disorder that promotes additional photon absorption, and (iii) increased film opacity caused by nanoparticle-induced absorption and scattering. The opacity increases dramatically from 0.343 A/mm for the pure film to 3.72 A/mm for the 0.15 wt% nanocomposite, confirming stronger light attenuation.

Overall, the incorporation of ZnO–Au NPs significantly improves the UV-blocking performance of the PVA/CMC films. The nanocomposites containing 0.10–0.15 wt% ZnO–Au exhibit the most effective UV shielding, reducing UV transmission to below 40% in the UVA region and below 30% in the UVB/UVC regions. These findings demonstrate that ZnO–Au NP incorporation effectively modifies the optical structure of the polymer blend, leading to improved UV attenuation and making the nanocomposite films promising candidates for UV-protective coatings and packaging applications.

The refractive index (*n*) was also estimated from the band gap values using the empirical relation *n* $=3.3668\;E_{g}^{-0.32234}$ [52], and results are presented in Table 2. A gradual increase in refractive index from 2.058 to 2.244 is observed with increasing ZnO–Au content, indicating higher optical density and stronger light–matter interaction within the nanocomposite films. Such materials with an elevated refractive index are advantageous for optical coatings and photonic applications due to their improved light management properties [53].

### 3.4. Thermal Analysis

**Figure 7:**
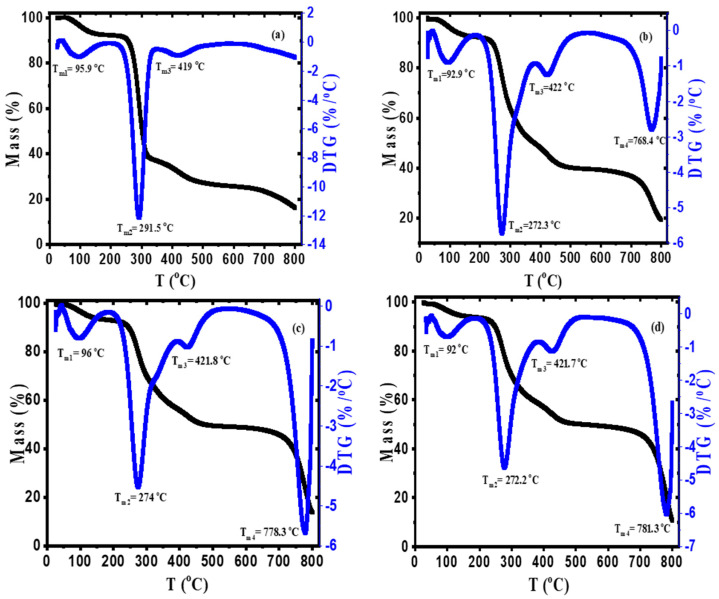
TGA/DTG plots of (**a**) PVA/CMC blend, (**b**) PVA/CMC/0.05 wt% ZnO-Au, (**c**) PVA/CMC/0.10 wt% ZnO-Au, and (**d**) PVA/CMC/0.15 wt% ZnO-Au.

Figure 7 displays the TGA/DTG curves of the pure PVA/CMC blend and the PVA/CMC/ZnO–Au nanocomposites. All samples were heated from 25 °C to 800 °C at a constant rate of ~10 °C/min. The pure PVA/CMC blend exhibits three distinct stages of thermal decomposition, whereas the ZnO-Au-incorporated nanocomposites undergo four stages. The maximum decomposition temperatures (*T*_m_) and corresponding mass losses for each stage are summarized in Table 3. The initial mass loss (6.08–7.93%) observed between 25 °C and 245 °C is attributed to the evaporation of physically adsorbed and bound water [54], as well as the removal of and the decomposition of hydroxyl groups. The second major stage, occurring between 245 °C and 375 °C, corresponds to the breakdown of side chains, cleavage of the polymer backbone, methylcellulose degradation, and CO_2_ release from the carboxylate (COO^−^) groups of CMC [55]. This finding aligns with earlier reports indicating rapid mass loss of CMC within the 300–400 °C range [56]. At higher temperatures (above 400 °C), a final mass loss of ~30–40% is observed, associated with further chain scission, dehydrogenation, and pyrolysis decomposition of the polymer matrix [57].

Overall, as shown in Table 3, the PVA/CMC/ZnO–Au nanocomposites exhibit lower mass loss across most degradation stages compared to the pure PVA/CMC blend, indicating improved thermal stability. The incorporation of ZnO–Au nanoparticles improves the thermal resistance of the PVA/CMC matrix, primarily as a function of the nanoparticle concentration. This improvement arises from two synergistic mechanisms: first, the ZnO–Au NPs interact with the polymer chains to form an inorganic–organic interfacial network that restricts polymer chain mobility and acts as a thermal barrier, thereby delaying the release of volatile degradation products [58]. Second, the thermal activation of ZnO generates oxygen species and oxygen vacancies within the nanocomposite framework. The liberated oxygen forms peroxyl radicals that facilitate controlled chain scission, while the oxygen vacancies act as electron traps and catalytic centers within the ZnO-Au domains. These combined effects play a crucial role in modulating the degradation kinetics and stabilizing the polymer matrix under thermal stress [59].

### 3.5. Surface Topography Analysis

**Figure 8:**
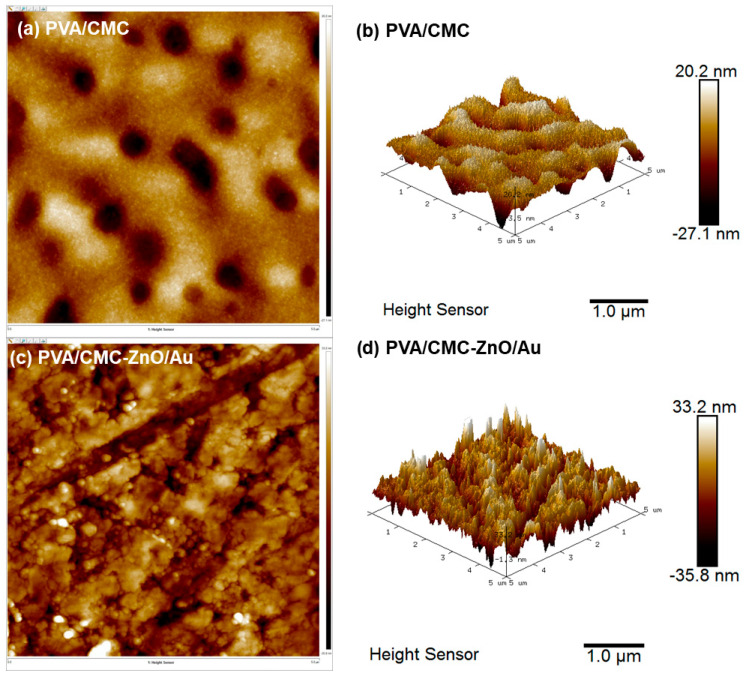
AFM images of (**a**,**b**) pristine PVA/CMC and (**c**,**d**) PVA/CMC-ZnO/Au (0.1 wt%) nanocomposite films.

The AFM images of the PVA/CMC and PVA/CMC–ZnO/Au nanocomposite films are presented in Figure 8a–d. The pristine PVA/CMC film exhibits a smooth and homogeneous topography with shallow surface undulations and low roughness values (*R*_a_ = 5.15 nm, *R*_q_= 6.68 nm, and *R*_max_= 39.2 nm). The alternating dark and bright regions represent minor height fluctuations, reflecting the hydrogen-bonded interpenetrating network typical of well-mixed polymer blends [54]. The absence of granular features indicates strong miscibility and molecular-level compatibility between PVA and CMC chains.

After incorporating ZnO–Au nanoparticles, as shown in Figure 8c,d the films exhibit more heterogeneous and textured morphologies, with roughness parameters increasing to *R*_a_ = 7.44 nm, *R*_q_ = 9.45 nm, and *R*_max_ = 60.5 nm for the PVA/CMC–ZnO/Au (0.1 gm) film. Numerous bright nanoscale protrusions appear across the surface, attributed to the embedded or partially exposed ZnO-Au nanostructures, which possess higher stiffness and electron density than the surrounding PVA/CMC phase [55]. The granular distribution of these bright spots and the absence of large aggregates suggest uniform NP dispersion and strong interfacial interactions with the PVA-CMC matrix, consistent with FTIR and SEM data. The introduction of ZnO-Au NP domains increases surface roughness and reactivity, favorable for bacterial membrane contact and reactive oxygen species (ROS) formation [56].

### 3.6. Antimicrobial Activity

**Figure 9:**
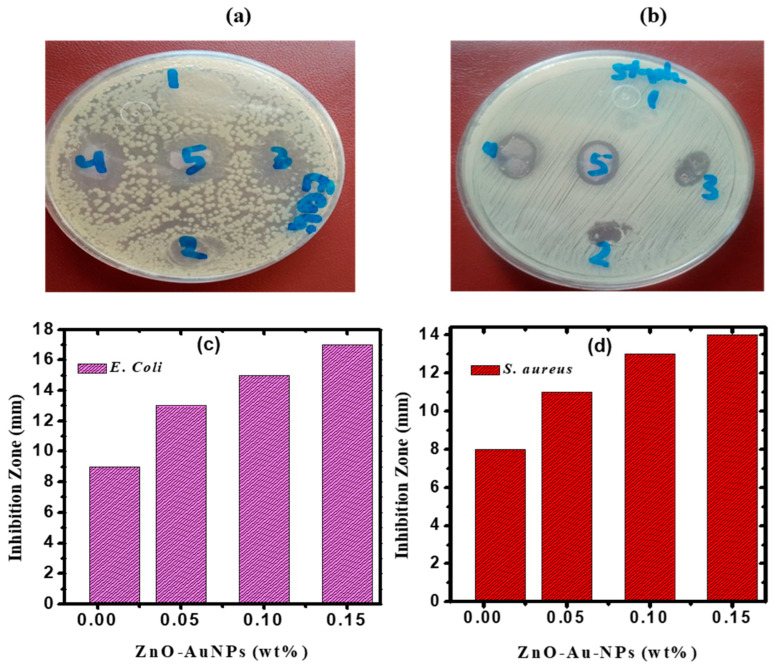
Disk diffusion images and inhibition zones of neat PVA/CMC and PVA/CMC–ZnO–Au films with varying nanoparticle contents against (**a**,**c**) *E. coli* and (**b**,**d**) *S. aureus*.

Figure 9a,b shows the disk diffusion assay results demonstrating the antibacterial performance of the pure PVA/CMC blend and the PVA/CMC/ZnO-Au nanocomposites against Gram-negative (*E. coli*) and Gram-positive (*S. aureus*) bacteria. The corresponding inhibition zone diameter, presented in Figure 9c,d, provides a quantitative evaluation of antibacterial efficiency. Incorporation of ZnO-Au NPs markedly enhanced the antibacterial activity of the PVA-CMC matrix, with the effect being more pronounced against *E. coli* than *S. aureus*. This enhancement becomes progressively significant with increasing ZnO-Au NP content, confirming that the nanocomposite efficiency is concentration dependent. that the nanocomposite’s antimicrobial activity is concentration dependent.

The superior bactericidal performance of the PVA/CMC/ZnO-Au nanocomposites arises from the synergistic action of ZnO-Au NPs embedded within the polymer matrix. ZnO contributes through the release of Zn^2+^ ions and generation of reactive oxygen species (ROS), which damage bacterial cell membranes and disrupt metabolic enzymes. The presence of Au enhances electron transfer and promotes localized surface plasmon resonance, intensifying ROS formation and improving interaction with bacterial membranes. The positively charged Zn^2+^ ions more readily penetrate the negatively charged outer membrane of Gram-negative *E. coli* than the thicker peptidoglycan-rich wall of Gram-positive S. aureus, explaining the higher inhibition observed for *E. coli.* The PVA–CMC matrix further facilitates uniform nanoparticle dispersion and controlled ion release, ensuring prolonged antimicrobial efficacy through combined physical disruption and oxidative stress mechanisms.

**Figure 10:**
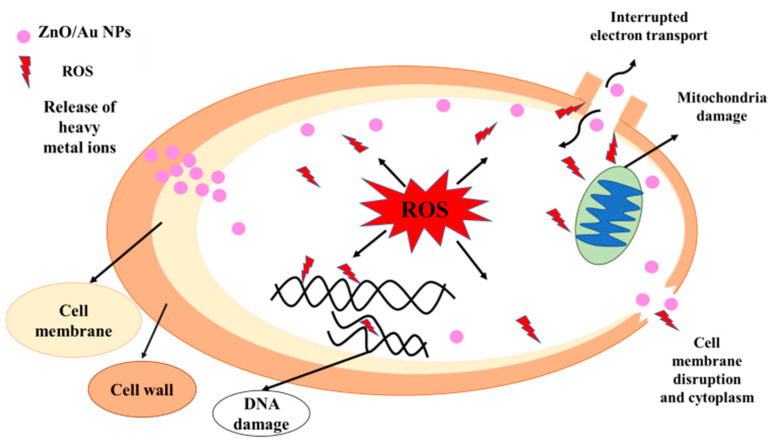
Mechanism representation of PVA/CMC-ZnO/Au nanocomposite films’ bactericidal activity.

As illustrated in Figure 10, ROS species, including superoxide anions peroxide (O^2−^), hydroxyl radicals (OH), and hydrogen peroxide (H_2_O_2_), play a major role in this process [60]. Their high oxidative potential destabilizes bacterial membrane potential and damages DNA, proteins, and lipids, leading to cell death [61]. The ZnO–Au nanocomposite generates higher ROS levels than ZnO due to the defect-rich ZnO lattice and enhanced charge transfer through Au incorporation [62,63]. These combined effects overwhelm antioxidant defense, resulting in irreversible oxidative damage and cell lysis.

## 4. Conclusions

PVA/CMC blend films incorporated with different weight percentages of ZnO–Au nanoparticles were successfully prepared using the solution casting method and characterized by XRD, FT-IR, Raman, XPS, and UV-Vis spectroscopy. XRD analysis confirmed the presence of characteristic ZnO diffraction peaks at 2θ = 32.13°, 34.79°, 36.64°, 47.86°, and 56.98°, along with a weak Au peak at 2θ = 63.34°. The results also indicated a gradual decrease in the crystallinity of the polymer matrix due to the increased amorphous nature and the formation of hydrogen bonding within the nanocomposite films.

FT-IR analysis revealed noticeable red and blue shifts in the O–H and C=O bands, confirming strong interactions between ZnO–Au nanoparticles and the PVA/CMC matrix through hydrogen bonding. Raman spectroscopy further verified the successful incorporation of ZnO–Au nanoparticles into the polymer blend. XPS analysis revealed the characteristic Au 4f and Zn 2p peaks, providing clear evidence of nanoparticle embedding within the polymer network and enhanced cross-linking interactions. High resolution C 1s and O 1s spectra also showed a reduction in C–OH and C–O groups, indicating stronger bonding interactions and improved cross-linking within the composite structure.

Optical analysis revealed a significant decrease in the optical band gap from 4.60 eV for the pure PVA/CMC blend to 3.52 eV for the film containing 0.15 wt% ZnO–Au nanoparticles. This reduction is attributed to the formation of localized electronic states within the band structure due to nanoparticle incorporation. Consequently, the nanocomposite films exhibited enhanced UV-shielding performance, where the UV transmittance decreased to approximately 24–29% in the UVC–UVB regions and about 38–39% in the UVA region, accompanied by a notable increase in film opacity.

Thermal analysis revealed improved stability, and antibacterial results confirmed that activity increased proportionally with ZnO–Au content. Overall, the synthesized PVA/CMC/ZnO–Au nanocomposites exhibit tunable optical, thermal, and antimicrobial properties, making them promising candidates for optoelectronic, coating, and antibacterial activity (Gram-negative and Gram-positive) strains.

Overall, the developed PVA/CMC/ZnO–Au nanocomposite films exhibit tunable optical, thermal, and antimicrobial properties. These features make them promising candidates for applications in UV-protective coatings, optoelectronic devices, and antibacterial materials against both Gram-negative and Gram-positive bacterial strains.

## Figures and Tables

**Table 1:** The average values of the structural parameters of the sample PVA/CMC/0.15 wt% ZnO-Au.

Parameter	Peak 1	Peak 2	Peak 3	Peak 4	Peak 5	Average
*D* (nm)	28.23	28.42	23.42	21.67	20.66	24.48
*ε*	4.64 × 10^−3^	4.25 × 10^−3^	4.91 × 10^−3^	4.11 × 10^−3^	3.67 × 10^−3^	4.32 × 10^−3^
*N_c_* (nm^−2^)	2.222	2.176	3.888	4.911	5.666	3.773
*δ* (nm^−2^)	1.25 × 10^−3^	1.23 × 10^−3^	1.82 × 10^−3^	2.12 × 10^−3^	2.34 × 10^−3^	1.75 × 10^−3^
*SF*	1.09 × 10^−2^	1.05 × 10^−2^	1.24 × 10^−2^	1.22 × 10^−2^	1.22 × 10^−2^	1.16 × 10^−2^

**Table 2:** The optical parameters of PVA/CMC/ZnO-Au nanocomposites.

Samples	Optical and Blocking Parameters						
	*E_U_* (eV)	*E_g_* (eV)	*T*_UVC (%)	*T*_UVB (%)	*T*_UVA (%)	Opacity A/mm	*n*
PVA/CMC	0.96	4.60	64.2	75.5	85.2	0.343	2.058
PVA/CMC/0.01 wt% ZnO-AuNPs	0.97	4.45	59.3	71.1	82.5	0.336	2.080
PVA/CMC/0.05 wt% ZnO-AuNPs	1.01	4.30	55.7	68.5	79.8	0.573	2.103
PVA/CMC/0.10 wt% ZnO-AuNPs	1.72	3.66	24.5	28.9	39.4	1.86	2.216
PVA/CMC/0.15 wt% ZnO-AuNPs	1.87	3.52	23.1	28.6	38.5	3.72	2.244

**Table 3:** Peak temperature (T_m_) and mass loss (%) for PVA/CMC and PVA/CMC/ZnO-AuO nanocomposites.

Sample	Stage I		Stage II		Stage III		Stage IV	
	*T*_m_ (°C)	Mass Loss (%)	*T*_m_ (°C)	Mass Loss (%)	*T*_m_ (°C)	Mass Loss (%)	*T*_m_ (°C)	Mass Loss (%)
Pure blend	95.9	7.64	291.5	55.17	419.0	20.31	----	----
S1	95.0	7.93	287.0	46.35	426.1	10.38	777.4	13.25
S2	92.9	7.62	272.3	42.75	422.0	9.96	768.4	20.36
S3	96.0	6.98	274.0	36.65	421.8	7.29	778.3	35.16
S4	92.0	6.08	272.2	35.24	421.7	5.71	781.3	39.33

## Data Availability

The authors confirm that the data supporting the findings of this study are available within the article.
